# Genetic instability-related lncRNAs predict prognosis and influence the immune microenvironment in breast cancer

**DOI:** 10.3389/fgene.2022.926984

**Published:** 2022-09-02

**Authors:** Zhenyi Lv, Qiang Wang, Xuxu Liu, Zhiwei Du, Wenping Liang, Tianming Liu, Yi Zheng, Biao Ma, Dongbo Xue

**Affiliations:** Key Laboratory of Hepatosplenic Surgery, Ministry of Education, The First Affiliated Hospital of Harbin Medical University, Harbin, Heilongjiang, China

**Keywords:** genetic instability, long non-coding RNAs, breast cancer, immune infiltration, keratinization

## Abstract

Genome instability is a hallmark of cancer, and the function of lncRNAs in regulating genomic stability has been gradually characterized. However, the prognostic value of lncRNAs related to genetic instability has not been found in breast cancer. Here we constructed a genetic instability-related lncRNA model including U62317.4, SEMA3B-AS1, MAPT-AS1, AC115837.2, LINC01269, AL645608.7, and GACAT2. This model can evaluate the risk and predict the survival outcomes of patients. Further analysis showed that the differentially expressed genes between the high- and low-risk groups were enriched in immunity and cornified envelope formation pathways. In addition, M2 macrophages infiltrated more obviously in the high-risk group. In summary, lncRNAs related to genetic instability may influence the development of breast cancer through immune infiltration and keratinization. This study provides a wider insight into breast cancer development and treatment.

## Introduction

Breast cancer is a heterogeneous disease, including prominent characteristics with specific pathologic features and biological behaviors (Simpson et al., 2005). According to the different lineage pathways, breast cancer can be divided into five subtypes: luminal A, luminal B, normal breast-like, HER2 overexpressing, and basal-like (Tang et al., 2008). Although the overall 5-years survival rate is high, some subtypes are still challenging to treat and have poor prognoses (Blows et al., 2010). A complex series of genetic changes causes breast cells to transform from precancerous lesions to carcinoma and affects the prognosis. Thus, it is necessary to estimate breast cancer prognosis by leveraging genetic changes.

Genome instability is a hallmark of cancer. Several mechanisms carefully preserve genomic integrity in normal cells, including DNA damage checkpoints, the DNA repair machinery, and the mitotic checkpoint. When these mechanisms are impaired, genomic alterations accumulate, leading to genome instability and driving cells from healthy to malignant (Duijf et al., 2019). In addition, genome instability gives rise to genetic heterogeneity, genetic diversity, and phenotypic diversity, facilitating tumor cell escape from immune surveillance and gradually developing into different subtypes. The different subtypes further lead to differences in prognosis. It has been demonstrated that genome instability can induce recurrence and therapeutic resistance in multiple myeloma (Neuse et al., 2020).

Genome instability includes the distinction between chromosomal instability or genomic instability and DNA instability or genetic instability (e.g., gene mutations) (Heng et al., 2013). The role of genetic instability (GI) in tumor development has been confirmed. BRCA1 can repair double-stranded DNA damage to maintain genomic stability, and BRCA1 mutations increase the risk of breast cancer (Welcsh and King, 2001). Due to GI, triple-negative breast cancer lacks the expression of epidermal growth factor 2, estrogen receptor, and progesterone receptor, which are common therapeutic targets (Ivanova et al., 2020). In luminal breast cancer, p27kip1, a necessary molecular for maintaining genomic stability, is often underexpressed (Berton et al., 2017). Therefore, early detection and intervention in GI may represent an effective measure for improving prognosis.

Various mechanisms can regulate GI. As one epigenetic regulatory molecule, lncRNAs play critical roles in genomic stabilization. LncRNAs are RNA molecules with transcripts longer than 200 nucleotides. They cannot be translated into proteins, but they regulate diverse cellular activities in normal development and disease occurrence by interacting with DNA, RNA, and proteins. LncRNAs regulate multiple signaling pathways in cancer development and metastasis (Peng et al., 2017) and are involved in regulating genomic stability. Noncoding RNA activated by DNA damage maintains genomic stability by binding to RNA-binding proteins (Lee et al., 2016). GUARDIN, a P53-responsive lncRNA, preserves genome integrity through TRF2 and BRCA1 (Hu et al., 2018). At present, genomic biomarkers are used to predict tumor prognosis in breast cancer research (Walsh et al., 2016), but whether lncRNAs related to GI predict survival outcomes of patients is still unclear.

Here, we divided samples from The Cancer Genome Atlas (TCGA) database into two groups. Based on the differentially expressed lncRNAs, we established a model to evaluate breast cancer prognosis. We explored the possibility of linking the lncRNA signature with GI and analyzed the pathways related to the lncRNA signature. Our results provide a new method for the early detection, screening, and treatment of breast cancer.

## Methods

### Data collection

Transcriptome, mutation, and clinical data of breast cancer were downloaded from TCGA database. The flowchart for the whole analysis is shown in Supplementary Figure S1.

### Screening lncRNAs associated with genetic instability and mRNAs co-expressed with these lncRNAs

The mutation frequency of breast cancer mutation data was analyzed using VarScan, and 986 samples were found to be mutated. The samples were sorted in descending order according to mutation frequency. Samples with the lowest 25% mutation frequency were regarded as the low mutation group (Low, *n* = 253), and those with the highest 25% mutation frequency were regarded as the high mutation group (High, *n* = 244). The lncRNAs of the two groups were extracted and analyzed. The average of multiple lines of a gene was displayed in only one line. The low expression lncRNAs (<0.4) were deleted. The Wilcoxon test was used to analyze the differences with a cutoff of |logFC|>1 and FDR <0.05. If logFC >1, the lncRNA was upregulated in the high mutation group, while if logFC < −1, the lncRNA was downregulated. These differentially expressed lncRNAs were defined as lncRNAs associated with genetic instability. Pearson correlation coefficients were computed to measure the correlation between differentially expressed lncRNAs and mRNAs. Correlation coefficient >0.2 and *p* < 0.05 were used as the criteria for screening mRNAs and the top 10 mRNAs with the strongest correlation were considered as the co-expressed mRNAs. GO function and KEGG pathway enrichment analyses were performed for the co-expression mRNAs.

### Classification of breast cancer

All breast cancer samples (*n* = 1,066) were clustered according to the expression of lncRNAs using the R hclust package for clustering. Then, the mutation frequencies of the two clusters were compared. A high mutation frequency indicated genetic instability, and a low mutation frequency indicated genetic stability.

### Data processing

For clinical data processing, samples with a follow-up time under 30 months were first deleted, and then samples with a survival time/survival status of 0 were deleted. For expression data processing, 122 differentially expressed lncRNAs related to genetic instability were extracted. Samples with intersecting clinical data and expression data were extracted, and then the clinical data and expression data were combined (all data = 998). We randomly divided the data into two sets (training set = 500, test set = 498). We performed univariate Cox and Lasso analyses on the data in the training set and screened out prognosis-related lncRNAs. Then, we constructed a prognostic risk model based on the following formula: $\text{Risk}\;\text{score}=\overset{n}{\underset{i=1}{\sum }}\text{coef}\;\left(\text{lncRNAi}\right)\times \text{expr}\;\left(\text{lncRNAi}\right)$ . The lncRNAi represents the ith prognostic lncRNA, expr (lncRNAi) is the expression level of lncRNAi for the patient, and coef (lncRNAi) represents the contribution of lncRNAi to prognostic risk scores that were obtained from the regression coefficient of multivariate Cox analysis. The median score of patients in the training set was used as a risk cutoff to classify patients into high- and low-risk groups. The test group and all data were analyzed using the same model formula as the training group, and the high-risk and low-risk groups were obtained.

The Kaplan-Meier method was used to calculate the survival rate and median survival for each prognostic risk group. The log-rank test was used to assess the difference in survival between the high-risk and low-risk groups with a significance level of 5%. Multivariate Cox regression and stratified analysis were used to assess the independence of the genetic instability-related lncRNA signature (GILncRNASig) from other key clinical factors. The hazard ratio (HR) and 95% confidence interval (CI) were calculated by Cox analysis. The performance of GILncRNASig was also evaluated by the time-dependent receiver operating characteristic (ROC) curve. All statistical analyses were performed using R version 4.0.2.

### Pathway enrichment analysis, screening of hub genes and the survival curve of related genes

Differentially expressed genes between the high- and low-risk groups were screened through the “limma” R package with a cutoff of |logFC|>0.5 and FDR <0.05. Pathway enrichment was analyzed through the Metascape website (http://metascape.org/) (Zhou et al., 2019). The cytoHubba and MCODE functions of Cytoscape were used to screen the hub genes and MCODE modules. The survival rates of the hub genes were downloaded from the Kaplan-Meier Plotter database. The sources for the database include GEO, EGA, and TCGA and the primary purpose of the tool is a meta-analysis-based discovery and validation of survival biomarkers (Lanczky and Gyorffy, 2021).

### Infiltration of immune cells

The CIBERSORT method was used to calculate the infiltration rate of each immune cell. Based on these results, we compared the infiltration rate between the high- and low-risk groups. CIBERSORT is an analytical tool to estimate the abundances of member cell types in a mixed cell population, using gene expression data (Newman et al., 2015). The association between immune infiltrates and gene expression was analyzed in the TIMER 2.0 database. This database is a comprehensive resource for systematical analysis of immune infiltrates across diverse cancer types (Li T. et al., 2020).

### Statistical analysis

Genetic difference analysis and the difference in immune cell infiltration between the two groups were assessed using the Wilcoxon test (Mann–Whitney). Survival analysis was performed using the log-rank test. A chi-square test was used to compare the frequency of TP53 mutations between the high- and low-risk groups. All data are expressed as the mean ± standard deviation (x ± s), ***p < 0.001, **p < 0.01, *p < 0.05.

## Results

### Screening of lncRNAs associated with genetic instability in breast cancer patients

**FIGURE 1:**
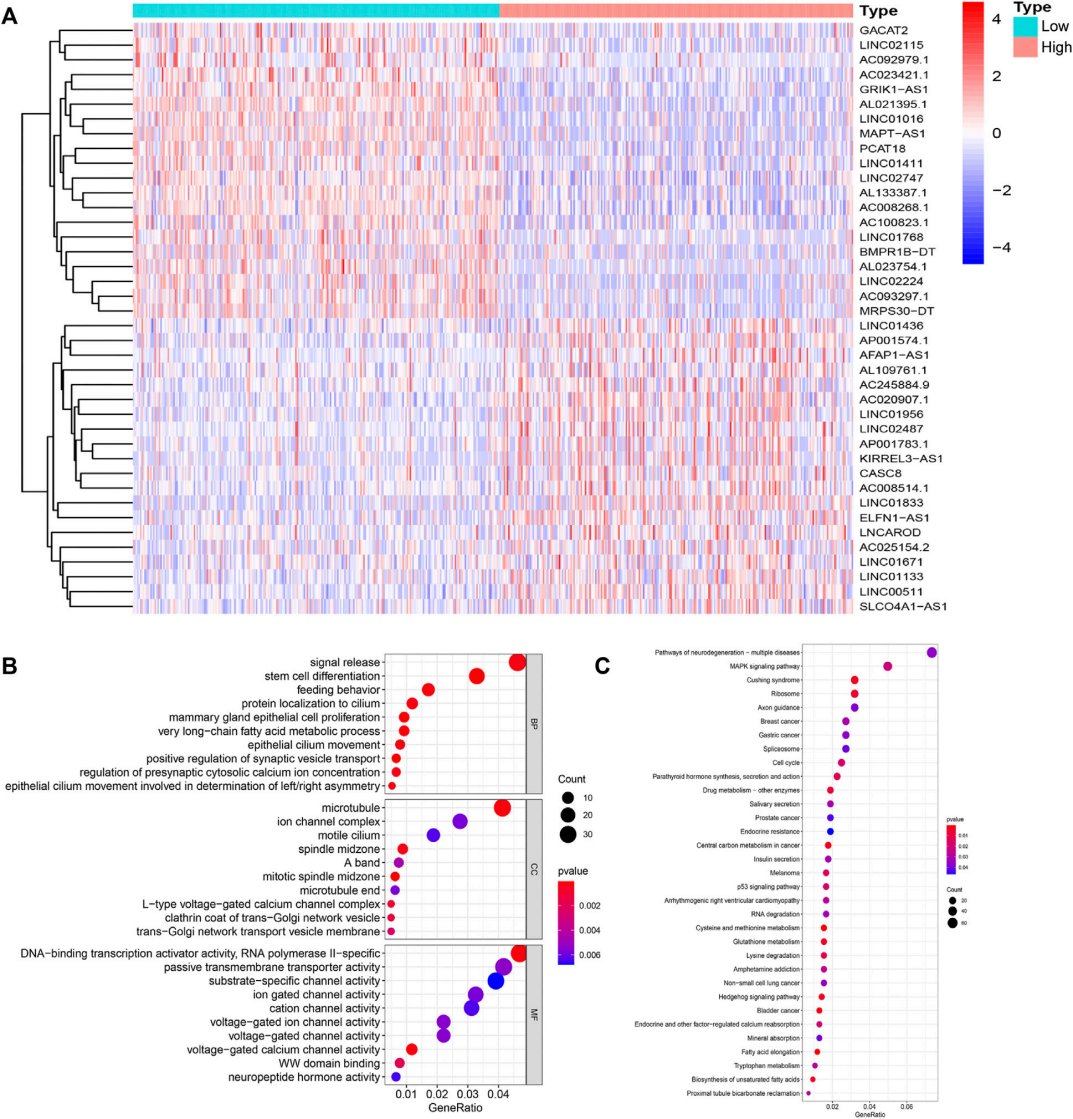
Identification and functional annotations of genetic instability-related lncRNAs in breast cancer patients. **(A)**. Differentially expressed lncRNAs between the High group and the Low group. Patients with the lowest 25% mutation frequency were regarded as the Low group (*n* = 253). Patients with the highest 25% mutation frequency were regarded as the High group (*n* = 244). |LogFC|>1 and FDR <0.05 were used as the criteria for screening differentially expressed lncRNAs. Red means upregulated and blue means downregulated. **(B)**. Functional enrichment analysis of GO for lncRNAs co-expressed mRNAs. **(C)**. Functional enrichment analysis of KEGG for lncRNAs co-expressed mRNAs.

To identify lncRNAs associated with GI, we calculated the cumulative number of genetic mutations in each patient and arranged them in descending order. The first 25% and last 25% of samples were divided into two groups. Based on the analysis, 122 lncRNAs were significantly differentially expressed. In the high mutation group, 55 lncRNAs were upregulated, and 67 lncRNAs were downregulated (Figure 1A). We defined these differentially expressed lncRNAs as lncRNAs associated with GI. According to the correlation between differentially expressed lncRNAs and mRNAs, we obtained mRNAs related to the lncRNAs (Supplementary Figure S2). Gene Ontology (GO) enrichment analysis revealed that these mRNAs were related to DNA transcription activity, ion channel activity, signal release, stem cell differentiation, and breast epithelial proliferation (Figure 1B). The KEGG pathway analysis showed that they were involved in the cell cycle, the MAPK signaling pathway, the p53 signaling pathway, and central carbon metabolism in cancer (Figure 1C).

### Breast cancer samples were divided into genomically stable and unstable subtypes

**FIGURE 2:**
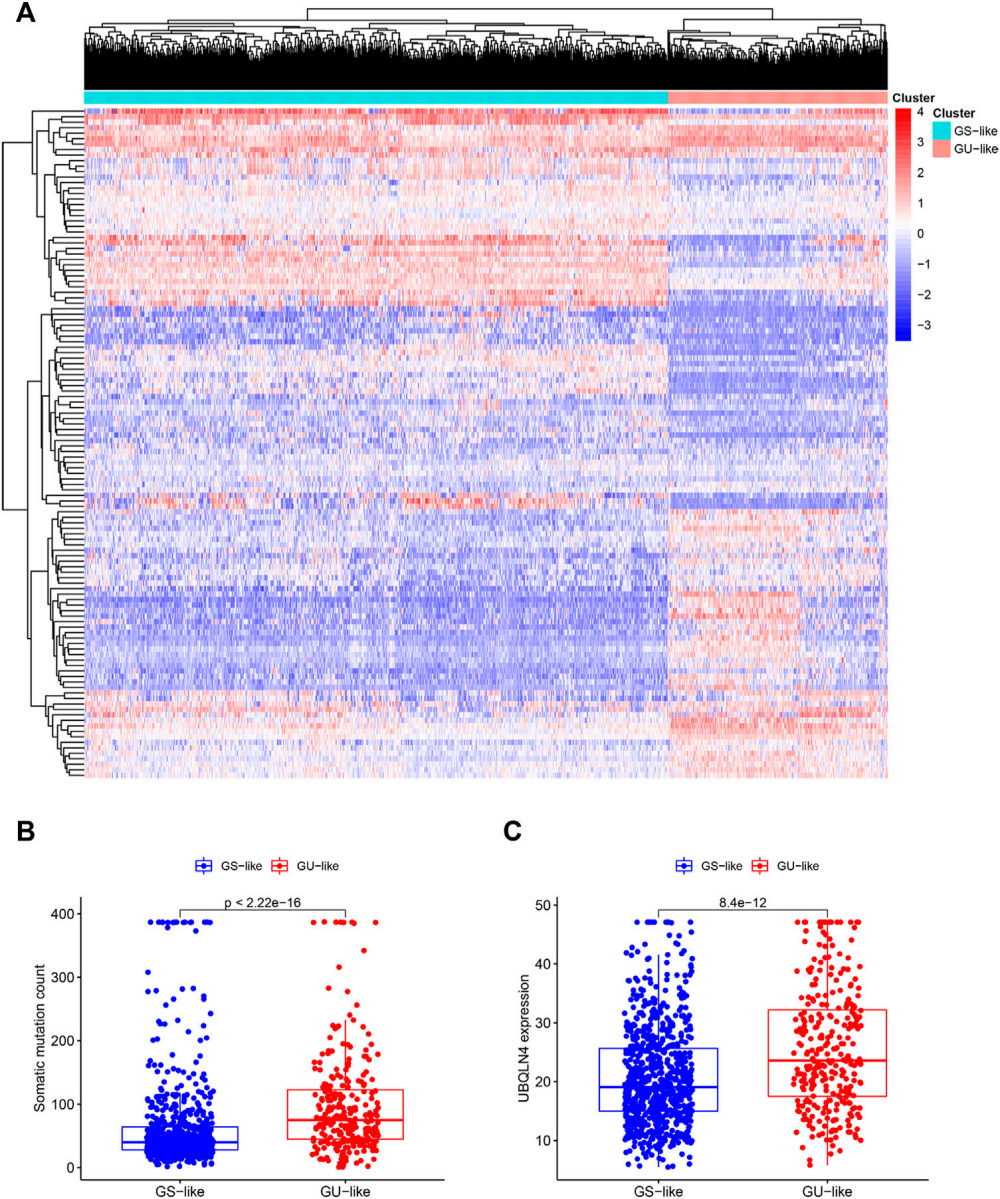
Hierarchical clustering of breast cancer patients and the characteristics between the groups. **(A)**. Hierarchical clustering of 1,066 breast cancer patients based on the expression pattern of 122 candidate genetic instability-derived lncRNAs. The left blue cluster was the GS-like group (*n* = 774), and the right red cluster was the GU-like group (*n* = 292). **(B)**. Boxplot of somatic mutations count between two groups. **(C)**. UBQLN4 expression level between these two groups.

Based on the differentially expressed lncRNAs, 1,066 breast cancer samples were divided into two clusters by hierarchical clustering (Cluster 1 = 774, Cluster 2 = 292). Comparing the mutation frequencies, we defined Cluster 1 as the GS group and Cluster 2 as the GU group (Figures 2A,B). Then, we compared expression levels of the UBQLN4 gene, a newly identified driver of genomic instability (Jachimowicz et al., 2019), between the GS and GU groups and found that the expression levels of UBQLN4 in the GU group were significantly higher than those in the GS group (Figure 2C).

### Construction of a genetic instability-derived lncRNA signature

**FIGURE 3:**
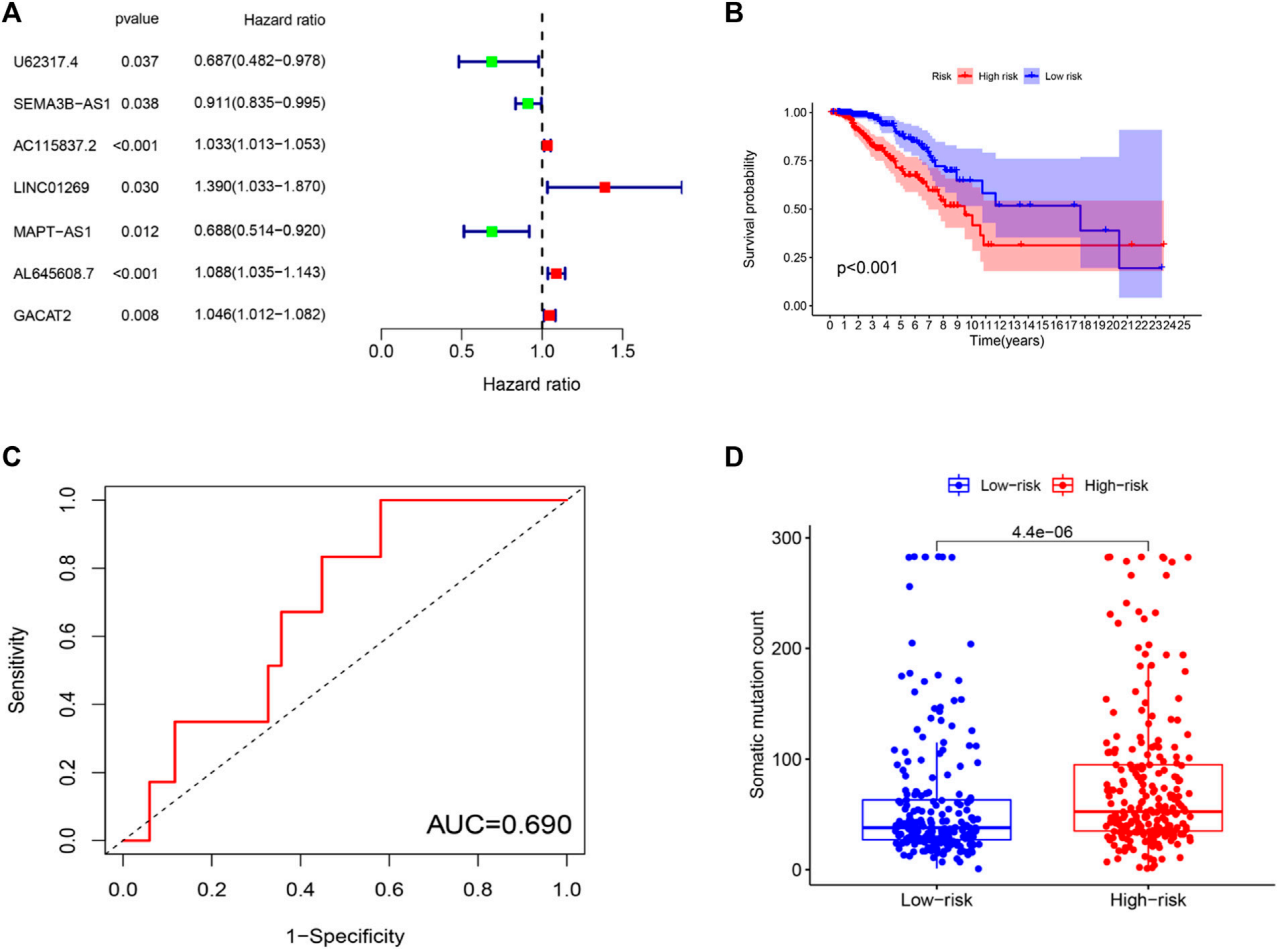
Establishment of genetic instability-related lncRNA signature (GILncRNASig) for prognosis prediction and identification of the predictive efficacy of the model. **(A)**. Forest plot of 7 lncRNAs, which were screened from candidate genetic instability-derived lncRNAs through univariate Cox analysis and Lasso analysis. **(B)**. Kaplan–Meier curves of overall survival of patients with low or high risk predicted by the GILncRNASig in the training set. **(C)**. Time-dependent ROC curve analysis of the GILncRNASig. **(D)**. Boxplot of somatic mutations count between the High-Risk and Low-Risk groups in the training set.

To further investigate the predictive function of these candidate lncRNAs, we screened 1,066 samples. We removed unqualified samples (those with a follow-up time under 30 months or survival time/survival status of 0) and ultimately obtained 998 samples (TCGA set). We randomly divided these samples into two sets (training set = 500, test set = 498). We ran statistical analyses on the clinical data comparing the groups to avoid intergroup differences (Table 1). The two groups were not significantly different based on their nongenomic stability-related characteristics. Using Cox regression and Lasso analyses, we compared the relationships between the expression of these 122 lncRNAs and overall survival, from which we constructed a seven-lncRNA model related to prognosis (Figure 3A). Then, we named the model GILncRNASig and used it to evaluate the prognostic risk (Supplementary Table S1). The coefficients of U62317.4, SEMA3B-AS1, and MAPT-AS1 were negative, indicating that these lncRNAs are low-risk lncRNAs and have a protective effect on clinical prognosis. In contrast, the coefficients of AC115837.2, LINC01269, AL645608.7, and GACAT2 were positive, indicating that they are high-risk lncRNAs and have a promoting effect on breast cancer. Next, we utilized GILncRNASig to obtain the risk score of each patient in the training set and divided them into high- and low-risk groups using the median risk (1.356) as the threshold. Kaplan-Meier analysis showed that the overall survival of patients in the low-risk group was significantly better (Figure 3B). Time-dependent ROC curve analysis showed the AUC of GILncRNASig was 0.690 (Figure 3C). We then classified patients according to their scores. We observed changes in the levels of GILncRNASig expression and somatic mutation counts with increasing scores (Supplememntay Figure S3A). For patients with high scores, the expression levels of the risk lncRNAs AC115837.2, LINC01269, AL645608.7, and GACAT2 were upregulated, while the expression levels of the protective lncRNAs U62317.4, SEMA3B-AS1, and MAPT-AS1 were downregulated. In contrast, GILncRNASig in patients with low scores displayed the opposite expression pattern. There were significant differences in somatic mutation patterns between the two groups, with the number of somatic mutations in the high-risk group being significantly higher (Figure 3D).

**TABLE 1:** Clinical characteristics of included patients.

Covariates	Type	Total (*n* = 998)	Train (*n* = 500)	Test (*n* = 498)	p value
age	≤65	724 (72.55%)	349 (69.8%)	375 (75.3%)	0.0606
	>65	274 (27.45%)	151 (30.2%)	123 (24.7%)	
gender	FEMALE	986 (98.8%)	494 (98.8%)	492 (98.8%)	1
	MALE	12 (1.2%)	6 (1.2%)	6 (1.2%)	
stage	Stage I-II	735 (73.65%)	370 (74%)	365 (73.29%)	0.7291
	Stage III-X	252 (25.25%)	123 (24.6%)	129 (25.9%)	
T	T1-2	840 (84.17%)	422 (84.4%)	418 (83.94%)	0.8505
	T3-4	155 (15.53%)	76 (15.2%)	79 (15.86%)	
M0	M0	827 (82.87%)	411 (82.2%)	416 (83.53%)	0.6905
	M1	21 (2.1%)	9 (1.8%)	12 (2.41%)	
N	N0-1	799 (80.06%)	400 (80%)	399 (80.12%)	1
	N2-3	182 (18.24%)	91 (18.2%)	91 (18.27%)	

### Independent validation of GILncRNASig in the test and TCGA sets

**FIGURE 4:**
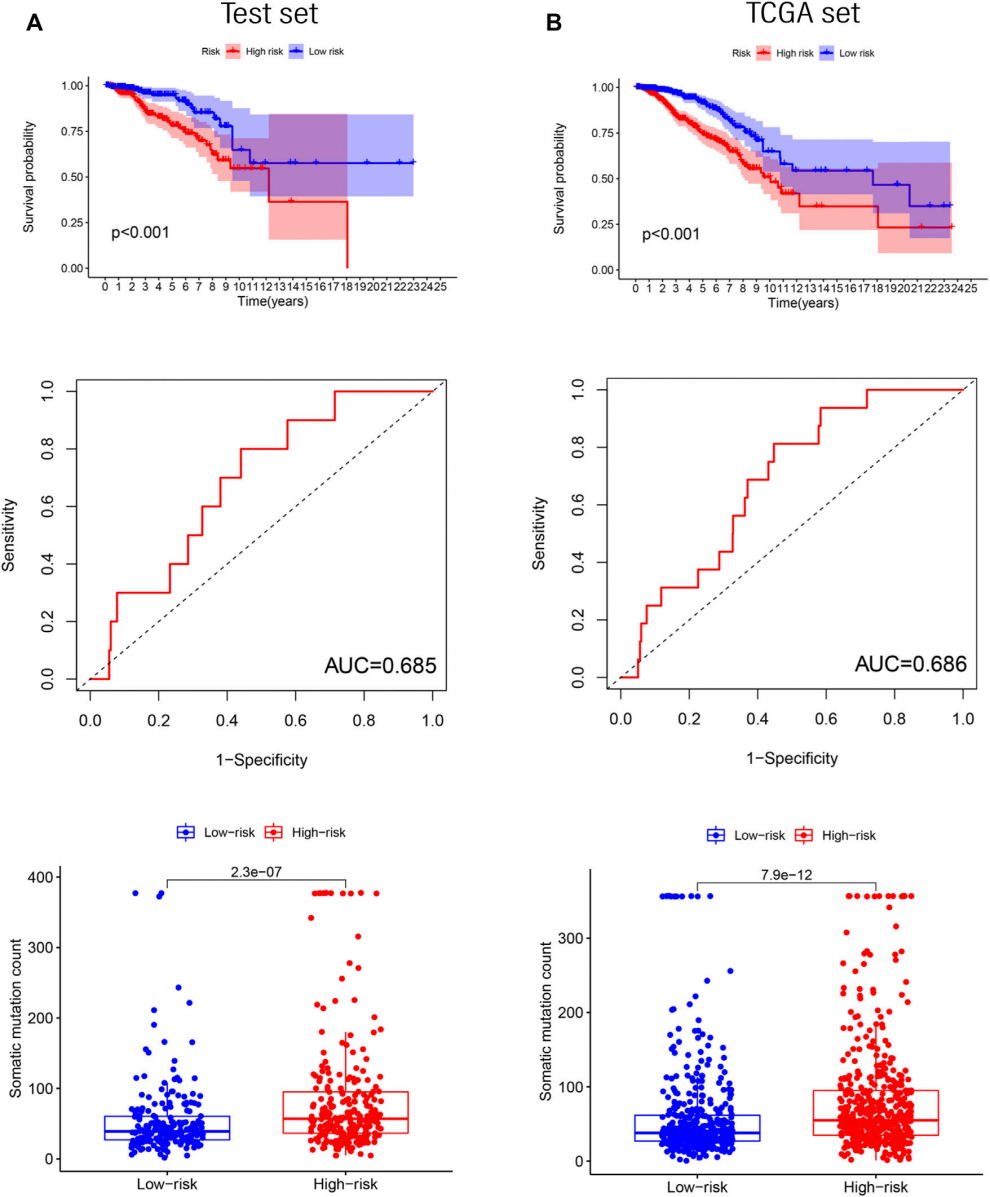
Verification of the GILncRNASig. **(A)**. The Kaplan–Meier curves and boxplot of somatic mutations between low- and high-risk, and the time-dependent ROC curve analysis in the testing set. **(B)**. The Kaplan–Meier curves and boxplot of somatic mutations between low- and high-risk groups, and the time-dependent ROC curve analysis in TCGA set.

To determine the accuracy of GILncRNASig, we tested its predictive performance in the other two sets. By applying the same risk model and threshold in the training set, 498 patients in the test set were divided into high-risk (*n* = 261) and low-risk (*n* = 237) groups (Figure 4A). Survival analysis showed that the overall survival of the high-risk group was significantly lower, and the AUC of GILncRNASig was 0.685. There were differences in somatic mutation count between the two groups. The expression of GILncRNASig in the test set are shown in Supplementary Figure S3A. The predictive performance of GILncRNASig in TCGA set was consistent with the above results. A total of 998 samples were divided into the high-risk (*n* = 511) and the low-risk (*n* = 487) groups (Figure 4B). The survival analysis showed that the overall survival of the high-risk group was lower with an AUC of 0.686. As the risk increased, the frequency of somatic mutations also increased. The expression of GILncRNASig in TCGA set are shown in Supplementary Figure S3A. TP53 is a crucial gene for maintaining genomic stability. We compared the frequency of TP53 mutations between the two groups. In the training set, 105 patients (42%) in the high-risk group had TP53 mutations, which was significantly higher than the 58 patients (23%) with TP53 mutations in the low-risk group (chi-square test *p* < 0.001). Similar results were also observed in the other sets (Supplementary Figure S3B).

### Performance comparison between GILncRNASig and other characteristic-related lncRNAs for survival prediction

**FIGURE 5:**
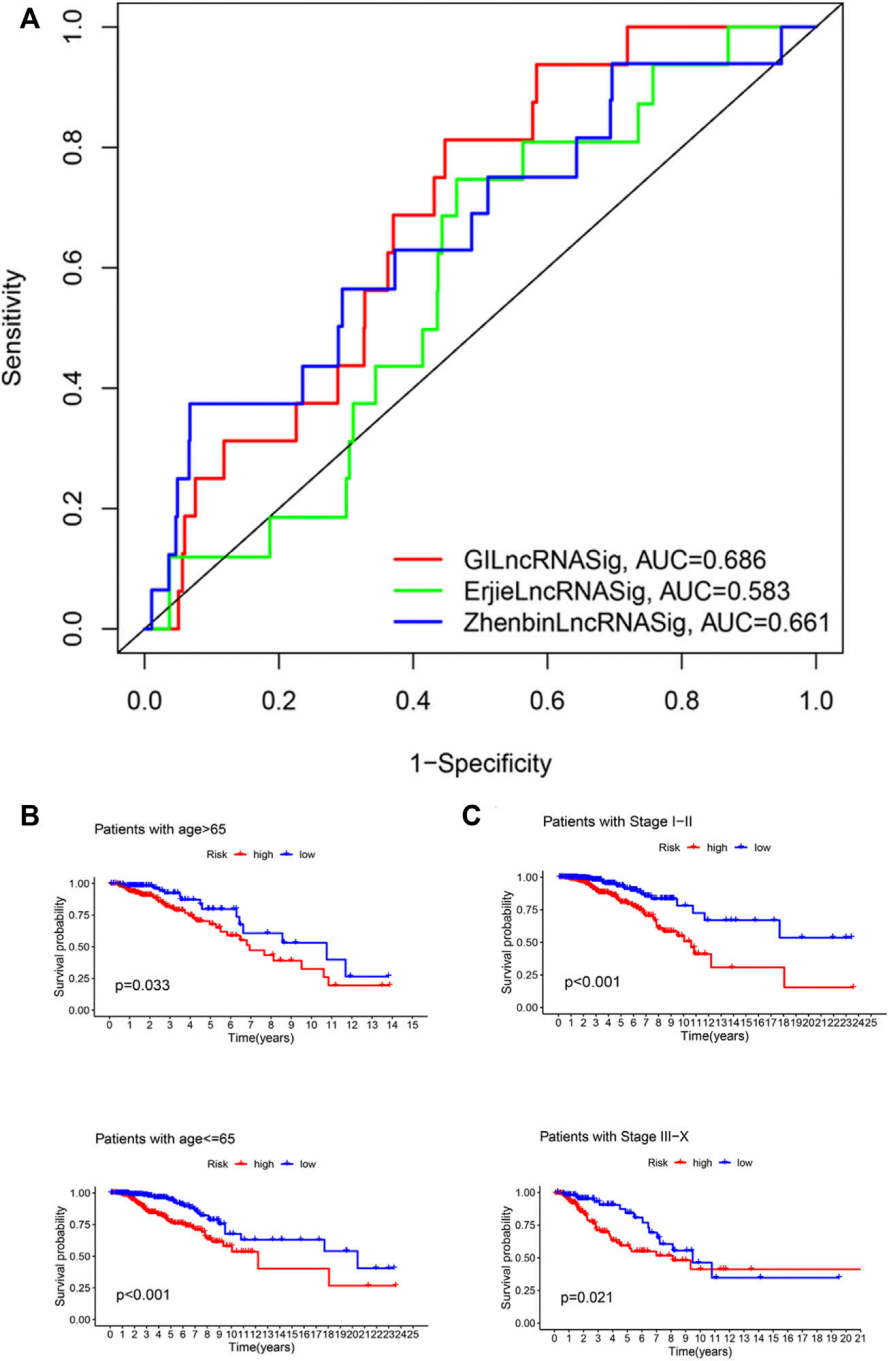
Identification of the efficiency of GILncRNASig in clinical characteristics. **(A)**. Comparison of the time-dependent ROC curve with other characteristic-related lncRNAs model. **(B)**. Kaplan–Meier curves of overall survival of patients between low- and high-risk groups in patients with Age <65 and Age ≥65. **(C)**. Kaplan–Meier curves of overall survival of patients between low- and high-risk groups in patients with Stage I–II and Stage III–IV.

We next compared the predictive performance of GILncRNASig to two recently published lncRNA signatures: the 6-lncRNA signature derived from Erjie’s study (hereafter referred to as ErjielncRNAsig) (Zhao et al., 2020) and the 8-lncRNA signature derived from Zhenbin’s study (hereafter referred to as ZhenbinlncRNAsig) (Liu et al., 2019). Using the same TCGA patient cohort, the AUC of GILncRNASig we constructed was 0.686 for 1-year OS, which was significantly higher than those of ErjielncRNASig (AUC = 0.583) and ZhenbinlncRNASig (AUC = 0.661) (Figure 5A).

### The efficiency of GILncRNASig in clinical characteristics

To assess the independence of GILncRNASig, we performed multivariate Cox regression analysis on age, pathological stage, and the predictive risk score model of GILncRNASig. Age and pathological staging were selected because they are important clinical factors affecting breast cancer prognosis. Breast cancer patients were divided into ages under 65 (*n* = 376) and over 65 (*n* = 419). They were further divided into high- and low-risk groups according to GILncRNASig. There was a significant difference in overall survival between the two groups (Figure 5B). Next, all breast cancer patients were stratified according to the pathological stage. Patients with pathological stages I or II were combined into the early-stage group (*n* = 593). Patients with pathological stage III or IV were combined into the advanced group (*n* = 181). GILncRNASig further divided the early-stage group into a high-risk group (*n* = 279) and a low-risk group (*n* = 314) and divided the advanced group into a high-risk group (*n* = 85) and a low-risk group (*n* = 96). The overall survival of the high-risk group was significantly lower than that of the low-risk group (Figure 5C). These results indicate the efficiency of GILncRNASig in clinical characteristics.

### GILncRNASig can be used to evaluate the immune infiltration in breast cancer

**FIGURE 6:**
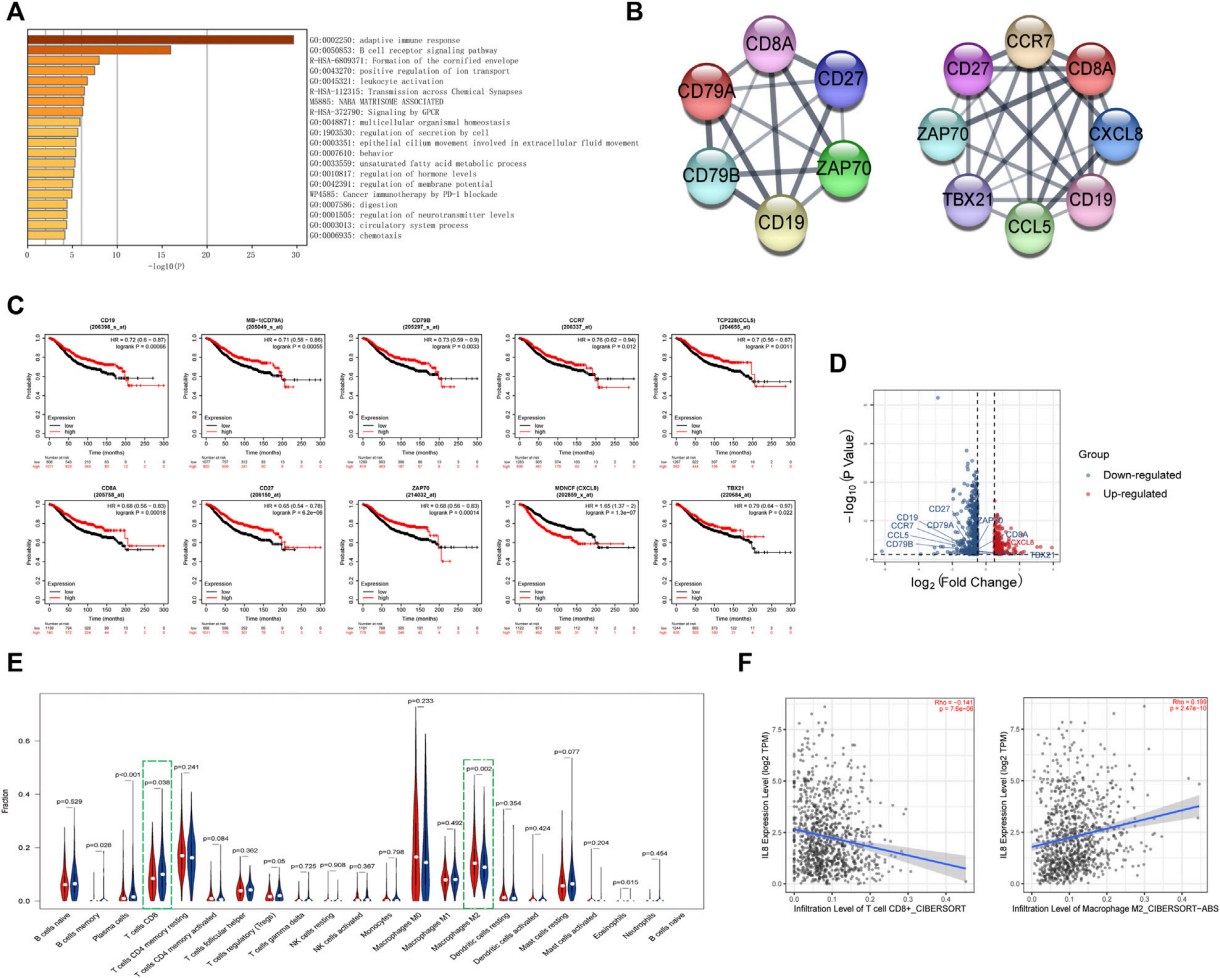
Identification of the immune-related pathways involved in the differentially expressed genes between high- and low-risk groups.**(A)**. The enrichment pathways for the differentially expressed genes between the high- and low-risk groups. **(B)**. The hub genes related to the adaptive immune pathway and the B-cell receptor signaling pathway. **(C)**. Kaplan–Meier curves of overall survival of the hub genes for breast cancer patients in Kaplan-Meier Plotter database. **(D)**. Volcano plot of hub genes between the high- and low-risk groups. The right red labeled gene was significantly higher in the high-risk group than the low-risk group, and the left blue labeled genes were significantly lower in the high-risk group than the low-risk group. **(E)**. Boxplot of the infiltration level of immune-associated cells in the high- and low-risk groups. **(F)**. The expression of CXCL8 was negatively correlated with CD8^+^ T cell infiltration and positively correlated with M2 macrophage infiltration in TIMER 2.0 database.

Next, we analyzed the differentially expressed genes between the high- and low-risk groups. The enriched pathways showed that these genes were related to various immune pathways, especially the adaptive immune response and the B-cell receptor signaling pathway (Figure 6A). Tumor-infiltrating immune cells are closely associated with prognosis prediction (Bense et al., 2017). By analyzing the protein-protein interactions, we screened six hub genes of the adaptive immune pathway and eight hub genes of the B-cell receptor signaling pathway (Figure 6B). Except for CXCL8 (MENCF/IL-8), the expression levels of other genes were down-regulated in the high-risk group, and correlated with better prognosis (Figures 6C,D). CXCL8 is a member of the chemokine family that induces the infiltration of immune cells. Therefore, we explored the differences in the immune microenvironment between the two groups. We found that CD8^+^ T-cells displayed a downward trend in the high-risk group, while M2 macrophages were increased (Figure 6E). We searched the association between immune infiltrates and the expression of CXCL8 from the TIMER 2.0 database. (Figure 6F). The results showed that CXCL8 expression was negatively correlated with CD8^+^ T-cell infiltration and positively correlated with M2 macrophage infiltration.

### GILncRNASig can be used to evaluate keratinization in breast cancer

**FIGURE 7:**
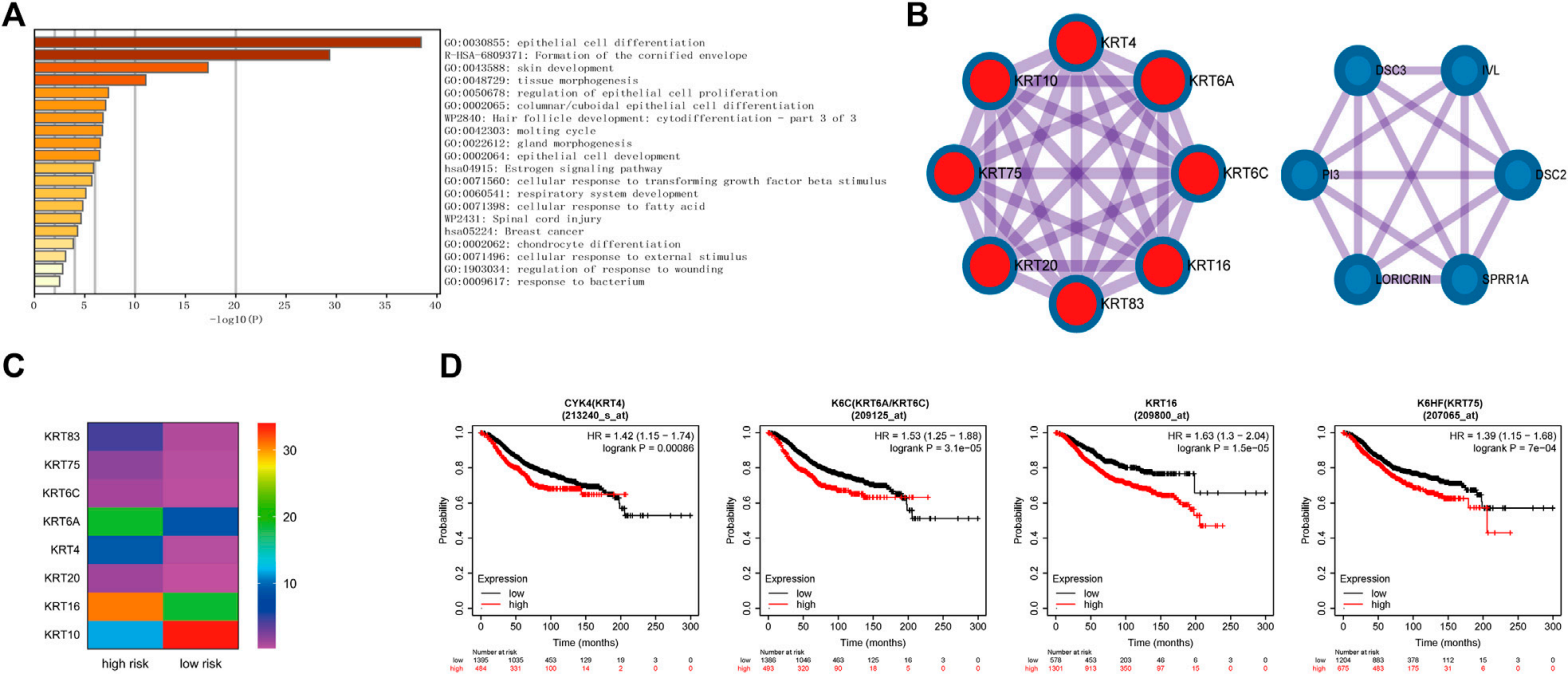
Identification of the formation of cornified envelope pathway involved in the differentially expressed genes between high- and low-risk groups. **(A)**. The enrichment pathways of genes involved in the formation of the cornified envelope pathway. **(B)**. The genes of two main MCODE modules analyzed by Cytoscape. **(C)**. The expression levels of genes in keratin-related MCODE module between the high- and low-risk groups. **(D)**. Kaplan–Meier curves of overall survival of some genes in keratin-related MCODE module for breast cancer patients in Kaplan-Meier Plotter database.

The previous analysis also revealed that the different genes were related to cornified envelope formation (Figure 6A). Further analysis of involved in this pathway, it indicated that these genes were primarily enriched in epithelial cell differentiation and formed two main MCODE modules (Figures 7A,B). One module was mainly composed of keratin family genes, most of which were upregulated in the high-risk group (Figure 7C). CYK4 (KRT4), K6C (KRT6A/KRT6C), KRT16, and K6HF (KRT75) affected overall survival (Figure 7D). In addition, the genes in another module also exhibited differential expression between the two groups and were related to prognosis (Supplementary Figure S4).

## Discussion

GI is reflected in various malignant tumors and precancerous lesions. Additional GI leads to different genetic lesions, ranging from increased point insertion and deletion frequency to chromosome rearrangement and ploidy changes (Burrell et al., 2013). With the accumulation of gene lesions, many molecules become abnormally expressed. Therefore, GI plays an essential role in tumor progression. LncRNAs have been proven to affect the expression of intracellular molecules, including genes relating to GI, through various mechanisms. Therefore, we screened lncRNAs associated with GI. These lncRNA target genes were enriched in various tumor related pathways, including MAPK signaling pathway, p53 signaling pathway, and central carbon metabolism. It is well known that changes in the p53 signaling pathway are closely related to tumorigenesis; the MAPK pathway plays a vital role in cell proliferation, differentiation, apoptosis, and tumor metastasis (Guo et al., 2020). In addition, central carbon metabolism is the main source of energy required. In normal cells, the tricarboxylic acid cycle provides energy for cell growth and development, while tumor cells need to induce the Warburg effect to satisfy consumption (Wong et al., 2017). This metabolic reprogramming is also closely correlated with GI (Yang et al., 2016). Thus, these lncRNAs participate in the regulation of tumor development.

Then, we screened lncRNAs related to prognosis from the above lncRNA set and constructed the GILncRNASig. It has been found that lncRNAs involved in the model participated in the occurrence and development of tumors. Among them, U62317.4 is related to autophagy (Li et al., 2021), and SEMA3B-AS1 is associated with the stemness regulation of breast cancer stem cells (Li X. et al., 2020). Overexpression of MAPT-AS1 is associated with a better prognosis in non-triple-negative breast cancer Wang et al., 2019). However, unexpectedly, the high expression of MAPT-AS1 is considered to promote tumor invasion, metastasis, and drug resistance in ER-negative breast cancer patients (Pan et al., 2018). In addition, LINC01269 is related to the prognosis of liver cancer (Liao et al., 2020), while GACAT2 influences the prognosis of gastric cancer (Tan et al., 2016). We verified the evaluation effect of GILncRNASig and demonstrated its independent predictive value.

According to the model, we divided the patients into two groups and compared the different genes. We found there were differences in immune pathways and M2 macrophage infiltration between the two groups. Tumor-associated macrophages, generally having an M2-like phenotype and function, contribute to tumor progression by accelerating angiogenesis, tumor cell activation, metastasis, and immunosuppression (Komohara et al., 2016). Immune cells are usually recruited by chemokines. CXCL8, as a hub gene in the pathway, is a member of the chemokine family. CXCL8 has been found to affect angiogenesis, tumor genetic diversity, immune escape, proliferation and metastasis, and multidrug resistance (Asokan and Bandapalli, 2021). In addition, tumor-derived CXCL8 can traffic M2 macrophages and mediate local immunosuppression (Zhang et al., 2020). Therefore, GI may affect the expression of CXCL8, leading to the change of M2 cell infiltration and finally influencing the tumor immune microenvironment.

Finally, we analyzed pathways related to forming the cornified envelope and found that they were closely associated with keratin. Keratin is a typical intermediate protein of epithelial cells that is highly specific to the epithelial type and cell differentiation stage (Moll et al., 2008). Keratin expression can affect epithelial cell migration and the ECM process (Yoon and Leube, 2019). Breast cancer is primarily derived from mammary epithelial cells. Many keratin genes have been found to affect the occurrence and treatment of breast cancer. Overexpression of KRT16 enhances cell motility and promotes breast cancer metastasis (Elazezy et al., 2021). KRT19 regulates cell proliferation and can predict the effect of CDK inhibitor treatment (Sharma et al., 2019). Targeting KRT1 may represent a new method for treating triple-negative breast cancer (Saghaeidehkordi et al., 2021). We speculate that GI may lead to abnormal expression of keratin genes and induce keratinization pathway activation in mammary epithelial cells, leading to the formation of breast cancer.

Although our study evaluates the degree of GI and predicts breast cancer prognosis, there are still some shortcomings. First, we could not conduct an in-depth analysis of all subtypes due to the limited data. Second, we need to verify GILncRNASig in additional independent datasets to ensure accuracy. Finally, we need to conduct further clinical and biological research on these seven lncRNAs to explore their role in the occurrence and development of breast cancer.

## Conclusion

In conclusion, we identified a GILncRNASig model to evaluate the prognosis of breast cancer patients. We also found that lncRNAs related to GI may affect keratinization gene expression and the immune microenvironment of tumors. These findings may provide wider insights into the development and treatment of breast cancer.

## Data Availability

The original contributions presented in the study are included in the article/Supplementary Material. Further inquiries can be directed to the corresponding authors.
