# Supplementary material for: Genetic instability-related lncRNAs predict prognosis and influence the immune microenvironment in breast cancer
Source: Front Genet. 2022 Sep 2;13:926984. doi: 10.3389/fgene.2022.926984 (PMC9478756; doi:10.3389/fgene.2022.926984)
Supplement: Supplementary file 1 [file Table1.DOCX]

**Table S1** The genome instability lncRNA signatures

| lncRNA | coef | HR | HR.95L | HR.95H | pvalue |
| --- | --- | --- | --- | --- | --- |
| U62317.4 | -0.623 | 0.536 | 0.353 | 0.815 | 0.004 |
| SEMA3B-AS1 | -0.075 | 0.928 | 0.844 | 1.020 | 0.019 |
| AC115837.2 | 0.036 | 1.037 | 1.018 | 1.057 | <0.001 |
| LINC01269 | 0.432 | 1.541 | 1.117 | 2.125 | 0.008 |
| MAPT-AS1 | -0.468 | 0.626 | 0.437 | 0.898 | 0.011 |
| AL645608.7 | 0.078 | 1.081 | 1.027 | 1.137 | 0.003 |
| GACAT2 | 0.064 | 1.066 | 1.025 | 1.108 | 0.001 |
