# Supplementary figures and images for: Genetic instability-related lncRNAs predict prognosis and influence the immune microenvironment in breast cancer
Source: Front Genet. 2022 Sep 2;13:926984. doi: 10.3389/fgene.2022.926984 (PMC9478756; doi:10.3389/fgene.2022.926984)

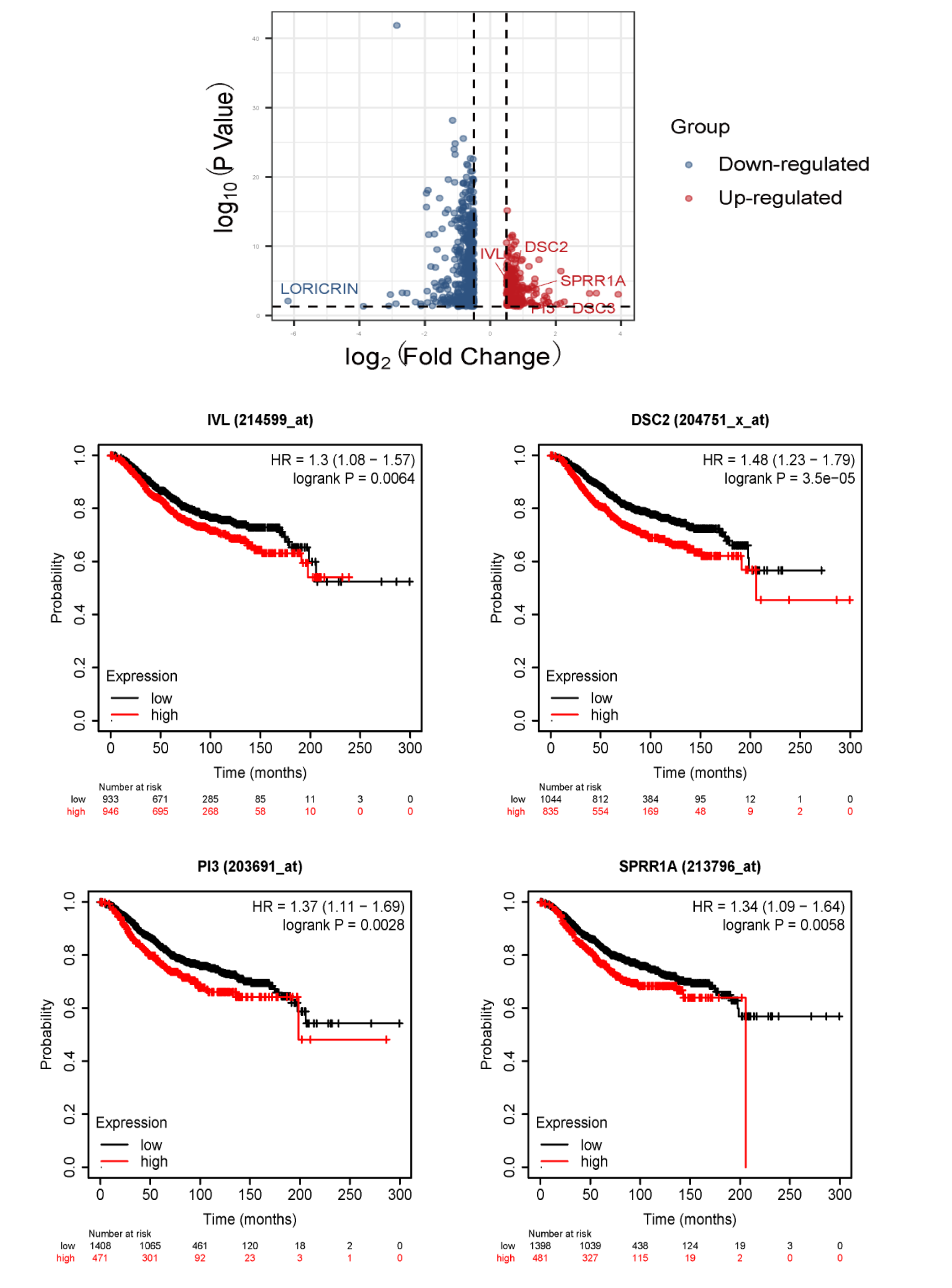

Supplement: Supplementary file 3 [file Image4.TIF]

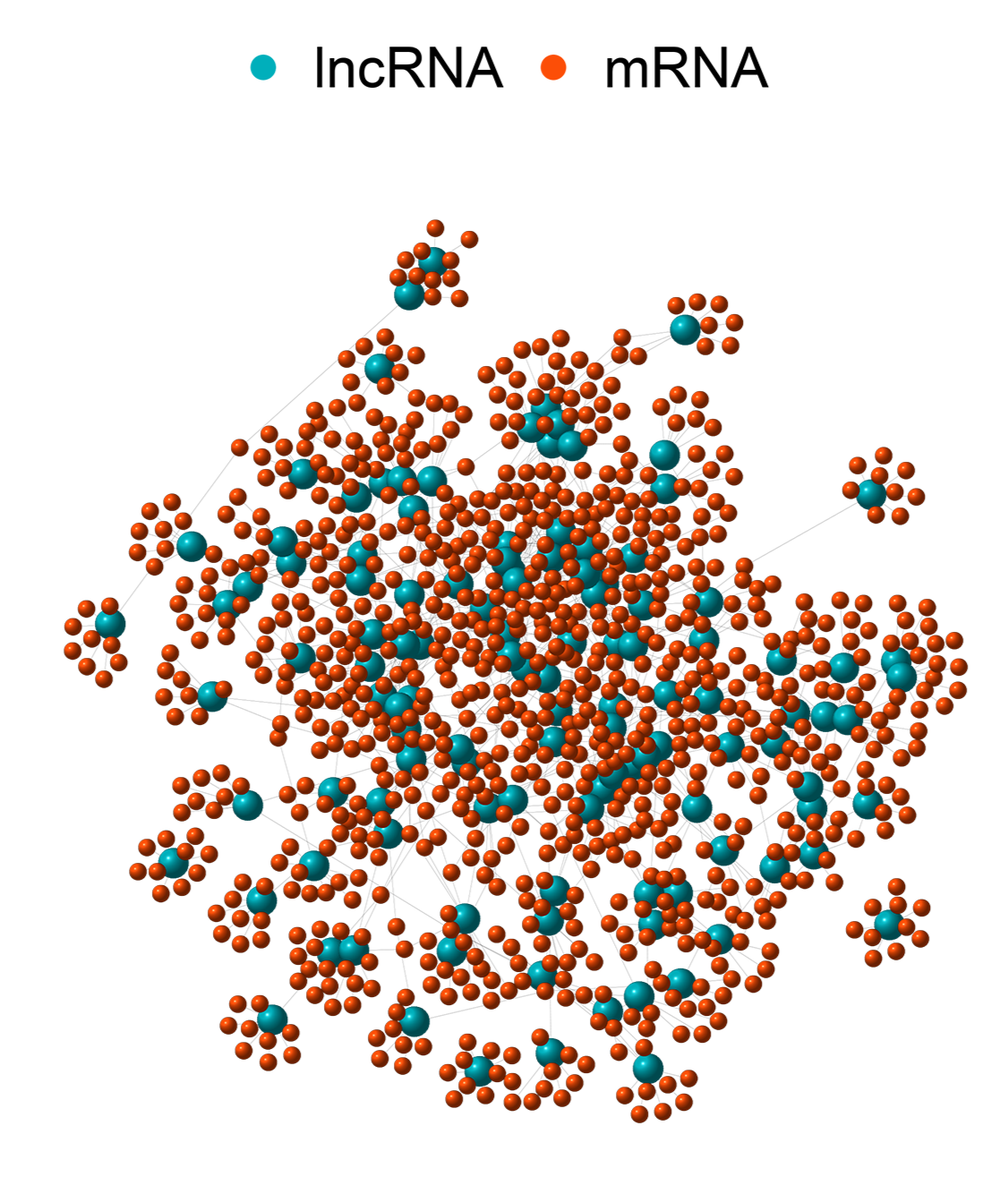

Supplement: Supplementary file 4 [file Image2.TIF]

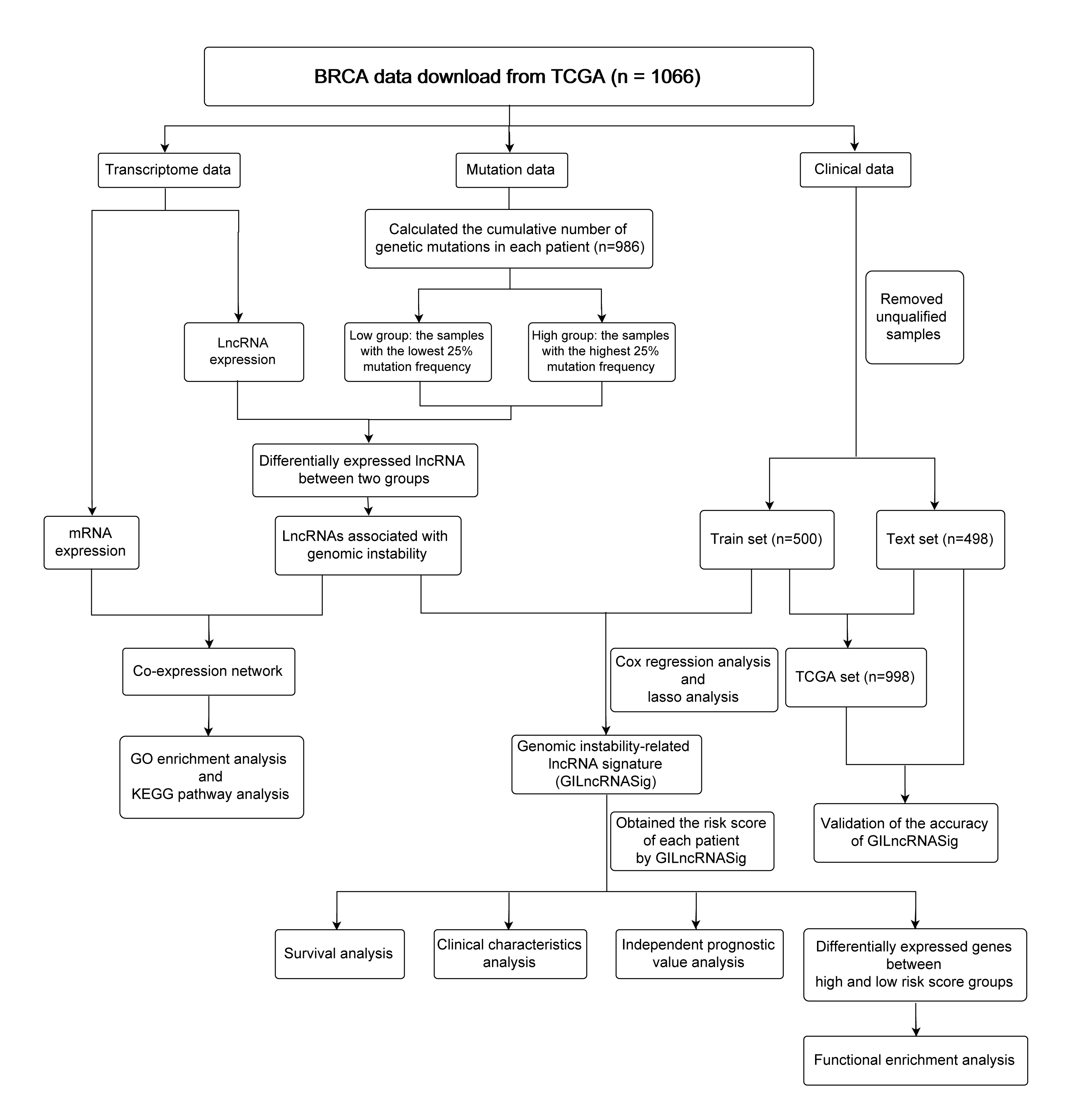

Supplement: Supplementary file 5 [file Image1.TIF]
